# Temperature and Resource Availability May Interactively Affect Over-Wintering Success of Juvenile Fish in a Changing Climate

**DOI:** 10.1371/journal.pone.0024022

**Published:** 2011-10-06

**Authors:** Jakob Brodersen, José Luis Rodriguez-Gil, Mikael Jönsson, Lars-Anders Hansson, Christer Brönmark, P. Anders Nilsson, Alice Nicolle, Olof Berglund

**Affiliations:** Department of Biology/Aquatic Ecology, Lund University, Lund, Sweden; University of British Columbia, Canada

## Abstract

The predicted global warming may affect freshwater systems at several organizational levels, from organism to ecosystem. Specifically, in temperate regions, the projected increase of winter temperatures may have important effects on the over-winter biology of a range of organisms and especially for fish and other ectothermic animals. However, temperature effects on organisms may be directed strongly by resource availability. Here, we investigated whether over-winter loss of biomass and lipid content of juvenile roach (*Rutilus rutilus*) was affected by the physiologically relatively small (2-5°C) changes of winter temperatures predicted by the Intergovernmental Panel on Climate Change (IPCC), under both natural and experimental conditions. This was investigated in combination with the effects of food availability. Finally, we explored the potential for a correlation between lake temperature and resource levels for planktivorous fish, i.e., zooplankton biomass, during five consecutive winters in a south Swedish lake. We show that small increases in temperature (+2°C) affected fish biomass loss in both presence and absence of food, but negatively and positively respectively. Temperature alone explained only a minor part of the variation when food availability was not taken into account. In contrast to other studies, lipid analyses of experimental fish suggest that critical somatic condition rather than critical lipid content determined starvation induced mortality. Our results illustrate the importance of considering not only changes in temperature when predicting organism response to climate change but also food-web interactions, such as resource availability and predation. However, as exemplified by our finding that zooplankton over-winter biomass in the lake was not related to over-winter temperature, this may not be a straightforward task.

## Introduction

In temperate freshwater systems, mean winter and spring water temperatures have been increasing during the last decades, most likely due to global warming (e.g. [1], [2]), and the regional climate projections by IPCC predict a further increase in mean annual air temperatures of 2 to 5°C until 2100 [3]. Recent climate change has led to a number of documented alterations of ecosystems throughout the world [4]. Climate change can be expected to affect organisms both directly, e.g., through temperature effects on consumption and metabolism, and indirectly through effects on trophic dynamics, such as resource availability and predation [4]. Research on all trophic levels, and interactions among them, is needed to achieve a mechanistic understanding of and make reliable predictions on the effects of climate change on ecosystem dynamics. Specifically, there is need for research that disentangles direct and indirect effects, e.g., temperature versus resource effects, as knowledge of their interactions is largely lacking.

The advanced mechanistic understanding of trophic processes and top-down effects in aquatic food-webs (e.g. [5], [6]) makes them ideal for studying climate change effects on various trophic levels in ecosystems. Previous studies on the topic have focussed on the dynamics of lower trophic levels, i.e., primary producers – phytoplankton, and primary consumers - zooplankton [7]–[10]. However, less research has been conducted on the effects of climate change on secondary consumers such as fish (see however [11]). This is unfortunate as especially young-of-the-year fish may strongly structure community and ecosystem processes in aquatic systems [12].

In Northern America, Europe and in Polar regions, mean temperatures are expected to increase more during winter than other seasons [13]-[16], suggesting that climate change effects may be strongest during winter. The winter period is of importance for fish population dynamics due to increased mortality risk (e.g. [17]). Among juvenile fish especially, a large part of the yearly mortality occurs during this period [18], [19], when their main food resource, zooplankton, occurs at low densities [20]. The abundance of zooplankton will, thus, partly determine the degree of starvation and thereby survival of juvenile fish during the winter period [21], [22].

Organisms use a number of strategies to survive the winter period, including building up energy stores, predominantly in the form of lipids, in autumn, as well as reducing activity during winter. Both of these strategies are found in fish [23], but also in other ectothermic animals such as invertebrates, reptiles, and amphibians [24]–[27]. But whereas many species of reptiles and amphibians hibernate during winter (e.g. [28]–[30], most fish maintain some degree of activity [28] and, thus, continue to consume resources during winter. Accumulation of energy stores may also serve to increase reproductive performance in the subsequent spring [23], [31], [32], and the rate at which energy stores are depleted during winter thereby affects not only over-winter survival, but also future reproductive output. The overwintering success of an individual can therefore be divided into primary success, i.e., survival, and secondary success, i.e., condition at the end of the winter.

In fish, as in other ectothermic animals, ambient temperatures influence lipid accumulation rates before winter [33], and depletion rates during winter [34], since enzymatic processes and basal metabolic rates generally increase with temperature. Temperature will, thus, affect individual risk of starvation [34]–[36] and thereby, potentially, population dynamics. For example, Reading [37] found a negative impact of mild winters on common toad (*Bufo bufo*) body weight and egg production, which was attributed to a higher metabolism during winter. As food consumption and assimilation rate are likely to drop with decreasing temperatures, most organisms may be challenged at the low temperatures during winter if intake- and/or assimilation rates are below the level needed for metabolism [38]. Many energetic models assume that consumption will drop to near zero at low temperatures leading to obligate starvation [39], [40]. However, this view has been seriously challenged by numerous studies (e.g. [38], [41]). Although not yet properly tested, it appears reasonable to assume that the utilization rate of lipid stores is affected not only by temperature, but also by food availability at very low temperatures. Research efforts that assess the response of juvenile fish to small low-temperature differences under different resource conditions are therefore much needed.

The aim of this study is to assess how differences in food availability in combination with small temperature differences affect secondary success, i.e., condition and total lipid content, of juvenile roach (*Rutilus rutilus*) during winter under laboratory conditions. The roach is a cyprinid species occurring in many lakes throughout most of Europe and Asia, from cold-water northern Scandinavian and Siberian- to warm-water Mediterranean lakes [42]. It commonly dominates lake fish communities, and is of key importance for structuring plankton communities in temperate lakes [43], [44]. Changes in the distribution and success of the roach due to climate change may thus have strong effects on lake dynamics. The laboratory results are compared with overwintering success of roach in the field. We hypothesize that at similar temperatures, fish without access to food will use a higher percentage of their lipid deposits than fed fish, but that there will be no effect of temperature on fish over-winter success when food is available. In presence of food, fish at all temperatures will be able to cover their metabolic demands, while unfed fish will use their deposits faster at higher temperatures, due to an increase of temperature-dependent metabolism. We further analyze a five-year data set on zooplankton density and water temperature from Lake Krankesjön to investigate if changes in zooplankton density, i.e. food availability for roach, are correlated to changes in winter temperature.

## Materials and Methods

### Capture of fish

Juvenile roach (*Rutilus rutilus*) from shallow Lake Krankesjön (average depth  =  0.7 m, maximum depth  =  3 m) in southern Sweden (55°42′N; 13°29′E; for lake description, see [12]) were caught on December 15 2005 with a dip net (1*1 meter; mesh size: 0.5 cm). All fish used in the experiment were between 39 and 61 mm in total length (51±4.0 mm, mean±SD) and were assumed to belong to the 0+ cohort, consistent with length-age data from survey fishing in the lake during autumn (J. Brodersen unpublished data).

A number of fish were used for measurement of length and wet weight (*n* = 86) directly after capture. A subset (*n* = 50) of these 86 fish were used for measurement of initial dry weight and a subset (*n* = 25) of these 50 fish were later used for initial lipid content. At the end of the study period (23 March 2006), fish were caught in the same area with electro fishing to compare development of the above mentioned parameters in fish from the natural population with development in experimental fish.

### Experimental design and sampling

Fish were kept at three different temperatures: 0.5, 2.5 and 4.5°C, resembling range of winter temperatures in Lake Krankesjön. Each temperature treatment consisted of six replicate barrels (45 L) with 40 fish in each. To half of the barrels, food was added, whereas the rest were kept without food. The fish in the food treatments were fed ad libitum with “Vitakraft® Koi Junior” pond fish food three times a day; excess food was removed. During the course of the experiment we sampled 15 out of the 40 fish in each barrel. Barrels were inspected every day for dead fish. During the first 110 days of the experiment only eight fish died in all barrels. However, between days 111and 113, eight fish died in the 4.5°C, treatments without food alone. This rapid increase in mortality led us to terminate the experiment.

Sampling of experimental fish took place once a week. One fish from each barrel was captured, weighed to the nearest 0.01 g (wet weight) and total length (*L_T_*) measured to the nearest 0.1 mm. Fish were then freeze dried to determine dry weight to the nearest 0.001 g. Condition of fish was calculated as Fultons Condition Index (*K* = *M L_T_^-3^*, where *M* is dry weight) based on dry weights. In many cases, condition is closely related to fish length due to allometric changes with fish size, and measures of condition should then be adjusted for fish length (e.g. [45]). The size range of fish used in this study was, however, relatively small and condition was not related to fish length (linear regression; *F* = 0.317; *p* = 0.574). We did therefore not adjust condition for fish length. Fish from every second sampling occasion, i.e. from every second week, were used for analysis of lipid content (see below).

In this study we used a repeated measures design with sampling of few fish at several time steps rather than having a large sample size but only end point values as common in other studies (e.g. [34]). This enabled us to detect differenced in lipid content and condition throughout the experimental period.

The study complies with the current laws in Sweden; ethical concerns on care and use of experimental animals were followed under permission (M14-04) from the Malmö/Lund Ethical Committee.

### Lipid analysis

The extraction and measurement of the total lipid content of the fish was carried out with a modified version of the Bligh & Dyer [46] method. Samples were freeze-dried for 3 days, their dry-weight determined and homogenized with a teflon pestle tissue grinder after cutting up the sample into small pieces. Thereafter, we added 7.5 ml of a modified Bligh & Dyer solution (dichloromethane/methanol/water in proportions 1∶2∶0.8) and after sonication samples were left overnight for extraction. After extraction, we added dichloromethane (2 ml) and Milli-Q water (2 ml) in order to reach the new proportions of 2∶2∶1.8 and get a two phase solution suitable for separation. The organic phase was separated by centrifugation and, after 3 washes of the sample with dichloromethane, the solvent was evaporated under flow of nitrogen (20°C) in pre-weighed tubes. Total lipid content was measured by weighing the tubes after evaporation and expressed as percentage of total fish dry-weight.

### Zooplankton and temperature monitoring

Zooplankton was sampled in Lake Krankesjön every second week from November to mid-April for five consecutive years, 2003–2008. Samples were taken at a fixed position at the deepest part of the lake. Ten liters of water were taken from the upper water column with a 1.2 m long Plexiglas tube with a diameter of 36 mm, filtered through a 45 µm net and preserved in Lugol's solution for further analysis in the laboratory. Zooplankton were counted on genus level with the exception of copepods, which were separated into cyclopoid and calanoid copepods. A subset of individual zooplankton from each taxon found in the sample were measured to nearest 0.05 mm and individual biomass were estimated from known taxa-specific length-weight relationships [47]. Total biomass was calculated by multiplying mean biomass with density for each taxon. Temperature was monitored at the outlet of Lake Krankesjön with an Onset StowAway Tidbit temperature logger every four hours from December 2003 to April 2008.

### Data analysis

For analyses of the development in body condition (*K*), and lipid content, we used deviations from the average among fish caught at the beginning of the experiment. The use of *K* in the following is, hence, referring to relative *K*. The development in *K* and lipid content was analyzed with repeated measures analysis in SPSS, with food availability and temperature as factors. The intercept was forced to zero as all values were relative to initial values. If significant, analyses were carried out for food and no-food treatments separately, with Tukey's post hoc test for between temperature effects.

Seasonal patterns in zooplankton density (Z) and temperature (*T*) from November to mid-April pooled over all years were analyzed with quadratic polynomial regression (ln *Z* = c + b*t* + a*t* ^2^ for zooplankton density and *T* = c + b*t* + a*t* ^2^ for temperature, where *t* is day of winter and a, b, and c are constants). Since both temperature and zooplankton density values are lowest during mid-winter and data were only analyzed over winter months, quadratic polynomial regression with a positive quadrant was used. Average residuals for each year were used as an estimate of yearly deviations of the average seasonal patterns in temperature and zooplankton biomass.

## Results

### Fish overwintering success

**Figure 1:**
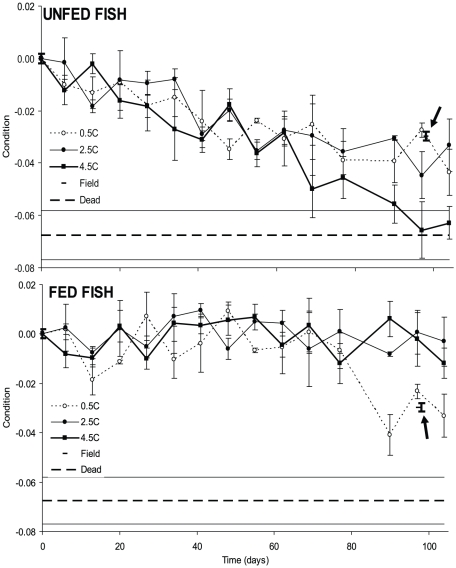
Standardized development of condition (*K*) for unfed (top panel) and fed fish (lower panel) at three different experimental temperatures. Values at day 0 are based on fish caught in the field and data for field caught fish at day 98 are indicated by arrows. Punctured horizontal lines indicate average (± S.E.) standardized dry-weight condition of fish that died during the experiment. Error bars indicate S.E. and bold error bars indicate S.E. for fish caught in the field at day 0 and day 98.

**Figure 2:**
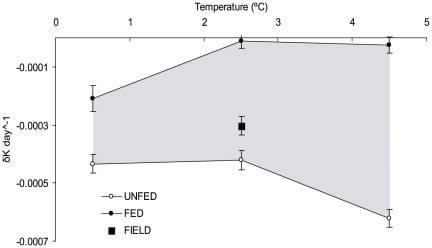
Average rate of change, calculated from regression coefficients, in relative body condition (δK day^−1^) for fed (filled circles) and unfed fish (open circles) at three different experimental temperatures. Filled square indicate fish in Lake Krankesjön, where the average winter temperature during the study period was 2.51°C and shaded area indicate the potential range for rate of change at different temperatures dependent on food supply. Error bars indicate standard error on regression coefficients.

Condition factor (*K*) was significantly affected by both temperature (rmANOVA; *F* = 10.4; *p* = 0.002) and food availability (rmANOVA; *F* = 390.7; *p*<0.001), and there was a significant temperature*food interaction (rmANOVA; *F* = 16.4; *p*<0.001). *K* decreased during the experimental period for fish in all treatments without food (Fig. 1), and there was a significant effect of temperature (rmANOVA; *F* = 309.8; *p*<0.001). Post hoc tests revealed that *K* in the 4.5°C treatment progressed significantly different from both the 0.5°C (Tukey HSD; *p* = 0.023) and the 2.5°C treatment (Tukey HSD; *p* = 0.011), whereas there was no difference between the 0.5°C and the 2.5°C treatments (Tukey HSD; *p* = 0.769). Thus, without food, *K* decreased at a faster rate in the warmest temperature (Fig. 2). At the end of the experiment, average *K* values in the 4.5°C treatment approached those of fish that died during the experiment (Fig. 1). Since mortality primarily occurred in the 4.5°C treatment towards the end of the experiment, the *K* value of dead fish (average *K* ± S.D. = −0.067 ± 0.025) could be seen as a critical value. Linear regression analyses suggest that this value will be reached after 108 days at 4.5°C; 160 days at 2.5°C and 155 days at 0.5°C. For each temperature, food had a significant effect on condition development (*p*<0.001 for all).

Temperature also had a significant effect on the progression of *K* (rmANOVA; *F* = 20.4; *p* = 0.001) in treatments with food (Fig. 1). The progression of *K* in the 0.5°C treatment was significantly different from both the 2.5°C (Tukey HSD; *p* = 0.004) and the 4.5°C treatment (Tukey HSD; *p* = 0.010), which were not significantly different from each other (Tukey HSD; *p* = 0.69). More specifically, *K* at 0.5°C was found to decrease during the experimental period (rmANOVA; test of within-subject contrasts; *F* = 29.5; *p* = 0.032), whereas there was no change in *K* over time in the two warmer treatments (2.5°C: *F* = 0.181; *p* = 0.712; 4.5°C: *F* = 0.008; *p* = 0.937; Fig. 1). However, the rate of decrease at 0.5°C was lower than any of the treatments without food (Fig. 2). A linear regression analysis showed that the critical *K* value would not be reached until after 324 days.

In the field population, condition was lower (*K* = −0.033) at the end of the study period than at the beginning (t-test; *t_73_* = 7.57; *p*<0.001). The average *K* in the natural population at the end of the experiment was lower than that of fed experimental fish, but higher than that of unfed fish (Fig. 1 & 2).

**Figure 3:**
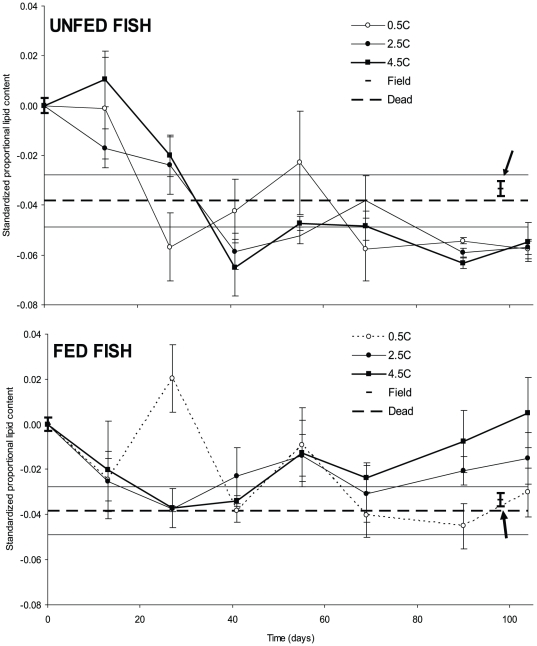
Standardized development of lipid content for unfed (top panel) and fed fish (lower panel) at three different experimental temperatures. Values at day 0 are based on fish caught in the field and data for field caught fish at day 98 is indicated by arrows. The average proportional lipid content of fish caught on day 0 was 0.1435. All lipid contents are given as deviations from this value. Punctured horizontal lines indicate average (± S.E.) standardized lipid content of fish that died during the experiment. Error bars indicate S.E. and bold error bars indicate S.E. for fish caught in the field at day 0 and day 98.

Food availability had a significant (rmANOVA; *F* = 10.2; *p* = 0.009) effect on the change in lipid content of fish, whereas neither temperature (rmANOVA; *F* = 0.40; *p* = 0.681) nor the food*temperature interaction (rmANOVA; *F* = 0.90; *p*  = 0.897) had any effect (Fig. 3). However, for unfed fish it appeared that lipid levels dropped to a relatively constant level and remained there throughout the experiment (Fig. 3), even before any mortality occurred in this treatment. Since the average relative lipid content of dead fish (average ± S.D  =  -0.038±0.028) was higher than the average end values for live fish without access to food, it did not appear that there was a critical value for lipid content (Fig. 3). Also in the natural population, the lipid content was found to be lower at the end of the study period than at the beginning (t-test; *t_48_* = 4.43; *p*<0.001; Fig. 3).

### Lake temperature and zooplankton dynamics

**Figure 4:**
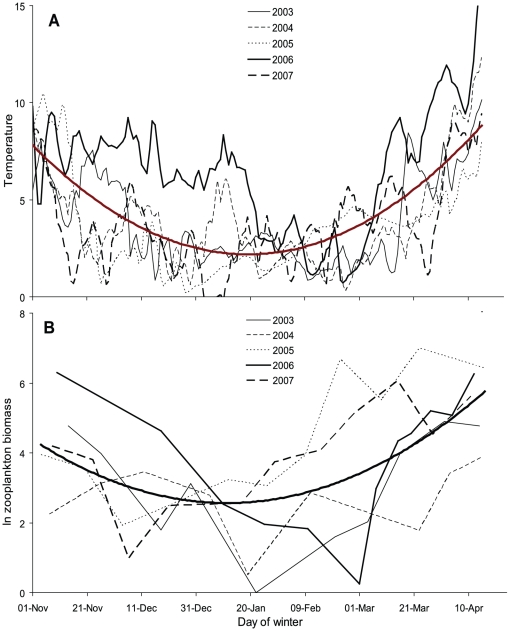
Seasonal development of (A) winter temperatures (°C) and (B) Ln zooplankton biomass (µgl^−1^) in Lake Krankesjön during five consecutive winters. Fat line in both figures refers to predicted values from quadratic polynomial regression analysis.

Variation in lake water temperature occurred both within and between years (Fig. 4a). In most years, water temperature dropped to below 2°C in mid-November (between November 14 and 19) except for the winter 2006/07, where lake temperatures below 2°C were not recorded until February 9. Temperature followed a quadratic polynomial pattern from November to mid-April (*T* = 7.5–0.15 *t* + 0.0009*t* ^2^; *r^2^* = 0.44; *p*<0.001) and yearly average deviation from this pattern ranged from -0.76 to +2.2°C. Hence, despite large within year variations, there were remarkable differences between years in winter water temperatures.

Zooplankton biomass and dynamics during winter, i.e., from November to April, varied considerably between years. For example, the highest zooplankton biomass (1087 µgl^−1^ on March 23, 2006) was more than three orders of magnitude higher than the lowest detected zooplankton biomass (0.3 µgl^−1^ on March 1, 2005). However, zooplankton biomass was generally low between early December and mid-February and the dynamics followed a quadratic polynomial pattern (ln Z = 4.5 -0.06*t* + 0.0004*t* ^2^; *r^2^* = 0.29; *p*<0.001) when including data from all years (Fig. 4b) and the average deviation from this pattern ranged between 54.2 µgl^−1^ below and 228.6 µgl^−1^ above the average.

Average deviation in zooplankton biomass during a winter was not dependent on the average deviation in lake temperature (linear regression, B = -0.016; *F* = 0.002; *p* = 0.968), i.e., years with low or high temperature were in general not associated with high or low densities of zooplankton.

## Discussion

Here, we have empirically shown that even small temperature differences (±2°C) may have significant effects on fish over-winter success, measured as change in body condition, both in the presence and absence of food. However, our results also clearly show that temperature is only of minor importance when food availability is not taken into account. Hence, increase in water temperatures during winter in the range predicted due to climate change may have consequences for fish survival. The effect may be influenced by the corresponding response of the food source, in this case the zooplankton community. Our field data from Lake Krankesjön did not suggest a winter density-temperature relationship for zooplankton. Only in very cold waters, where water temperature is close to freezing during longer periods in winter, may an increased temperature have a positive effect on the fish community, but then only if food is available.

In our laboratory experiment, we found no significant difference in condition or lipid content between the 2.5°C and the 4.5°C treatment where food was available. Differences are not expected at the higher range winter temperatures, as most temperate fish do not grow at any temperature found during winter, e.g., for roach below 12°C [48]. However, in a situation where food is limiting, the increased winter temperature will most likely have negative impacts on fish condition, dependent upon the amount temperature increases. For example, at low food levels, a two degree increase from 2.5°C to 4.5°C has a larger effect than an increase from 0.5°C to 2.5°C. Our results thus suggest that climate warming is likely to have different effects on fish over-wintering success depending on local temperature regimes. If local winter temperatures are originally very close to 0°C, a few degrees warming may be beneficial for the fish, but if local winter temperatures are originally above 2°C, a further increase of water temperature during winter may have a negative impact on fish over-wintering success. Together, these notions indicate that the effect on climatic winter warming on the fish community depends on the food supply, and that the potential benefits from increased winter temperature are much smaller than the potential risks. This may not only apply to fish, but also to other non-hibernating ectothermic organisms living in water, such as molluscs, arthropods and crustaceans, when winter temperatures are below the minimum temperature that allows somatic growth. However, before generalizing the results to other species, it should be taken into account that fish, as other ectothermic organisms, have different temperature optima and different adaptations to low temperatures [49], [50]. Roach may be better at coping with low temperatures than e.g. more warm-water adapted species such as many North American centrarchids, European tench (*Tinca tinca*) or crucian carp (*Carassius carassius*). For these more warm-water adapted species, food availability may not have a large influence at our lowest temperatures, although the general conclusions of this study should still apply at their higher temperature ranges. On the other hand, cold-water adapted fish such as salmonids, especially Arctic charr (*Salvelinus alpinus*) can be hypothesized to be even more vulnerable to higher winter temperatures (e.g. [51]).

Food availability had an impact on condition development even at the lowest temperature treatment (0.5°C), which is in line with observations of roach feeding during winter in similar ecosystems [52]. This illustrates that food intake is important for overwinter success, i.e., decreasing depletion rates of lipid reserves, even at temperatures very close to freezing. This further supports past research questioning the obligate starvation at very low temperatures due to minimum food intake [41], although the reduction in fish condition at 0.5°C when food was available is noteworthy. We hypothesized that temperature would not affect fish condition when food was available, as fish at all temperatures would be able to adapt their food intake to their metabolic demands. However, these results indicate that fish may not assimilate food at rates that correspond to their metabolic rates at this temperature. Irrespective of the cause, the drop in condition may have effects on individual fish and on fish communities. It is unlikely that this will lead to death by starvation during winter as it would take almost a year to reach the critical value for *K*.

Previous studies have suggested critical or threshold lipid levels for fish, below which overwinter survival will be affected [34], [53]. In our study the minimum lipid levels reached in fish did not indicate a threshold level regarding survival, as mortality was low, and surviving fish reached levels lower than the few fish that died during the experiment. Minimum levels were reached early in all unfed treatments, around day 40 (Fig. 3), and remained at this level during the remainder of the experiment without significantly affecting survival in these treatments, while condition factors decreased continuously throughout the experiment. This implies that fish used other energy sources than lipids in the unfed treatments, e.g., white muscle protein or glycogen [54]. Lipid levels in fish in the field decreased during the experimental period; however they did not approach the low levels found in the unfed treatments. Although starvation-induced mortality in our study was low, the different effects on condition and lipid levels caused by temperature regime and food availability may still impact populations in the field. The regression models did not predict significant starvation-induced mortality in the field fish. However, different environmental condition during the growth season in other years may create different before-winter sizes of fish (e.g. [55]). In fact, from survey data from the lake, we see years with both a significantly higher and significantly lower average size of 0+ roach during autumn (J. Brodersen unpublished data), which could lead to starvation mortality in years where 0+ roach are relatively small. Many animals are able to compensate for periods of food deprivation by subsequently increasing growth rates above those of non-deprived animals [56], [57]. However, this compensatory growth comes with a cost, e.g., increased predation caused by risk-taking behaviour or increased delayed mortality [11], [58]. Thus, these sub-lethal climate and food induced differences in fish condition and lipids may have profound effects on fish population dynamics and food web interactions in the field.

Our analysis of five years of zooplankton and temperature data from Lake Krankesjön showed that there was no correlation between lake temperature and zooplankton biomass during winter despite high among year variation. Although this does not exclude that zooplankton biomass during winter will be affected by climate change (see e.g., [59]), it indicates that the zooplankton response is complex and not directly coupled to temperature, and that it makes sense to treat temperature and food availability as two independent explanatory variables for fish over-wintering success. This further illustrates the importance of increased knowledge on how zooplankton winter biomass will develop under different climate change scenarios. At present, most focus has been on the effects on zooplankton spring phenology. Here zooplankton biomass is thought to increase earlier in spring with the increase of winter and spring mean temperatures [10], [60]-[62], although also mismatch scenarios, where spring phenology of zooplankton does not follow temperature changes, have been suggested [7], [63]. Although an early zooplankton peak may save some fish from starvation, it is of little importance if fish reach critical *K*-levels prior to the zooplankton increase. This is illustrated by the prediction from the linear regressions obtained in our study, where fish at 4.5°C without access to food would reach a critical condition around April 1, whereas for fish at lower temperatures this would not happen until mid-May. Although the lake temperature data suggests that winters with 108 days of temperatures of 4.5°C may be rare, there is still reason to believe that fish may end up suffering from the higher winter temperatures. First of all, temperatures above 4.5°C during seasonal cooling and warming of lakes may increase the rate of condition loss if sufficient food is not available. Secondly, fish may in some years have a lower condition when entering the winter period due to environmental conditions during the growth season (e.g. [55]), which may lead to a shorter time to reach critical condition levels. Furthermore, other lakes may have longer winter periods, and in deep lakes fish may stay close to the bottom [64] at temperatures closest to our warmest treatment. Fish from the field population were still far from the critical *K* value at the end of March. This could be explained by relatively normal temperatures, but higher than average zooplankton densities. In the five year data set, there was potential for up to a 2°C higher average temperature coinciding with lower winter zooplankton density, which would lead to a faster decrease in body condition, and could result in fish reaching critical *K*-levels.

Whereas our laboratory experiment included only treatments with either excess food or no food, fish will under natural conditions most often experience resource availabilities in between these extremes. The data on zooplankton biomass in the lake does suggest, however, that food availability for zooplanktivorous fish may range between very low to excess food availability. This is best exemplified by the extremes in zooplankton biomass in March with zooplankton biomass only just above detection level (0.3 µg l^−1^) in one year and very high zooplankton biomass (1087 µg l^−1^) in another. To further increase our understanding of when food availability becomes limiting under field conditions during winter (e.g. [18]), there is a need for studies quantifying critical resource densities for zooplankton feeding fish at winter conditions.

In our experiment, we kept all treatments at constant temperatures to limit the number of independent variables. Under natural conditions, however, characteristics in the cooling and warming before, during and at the end of winter may have significant impacts on overwintering success of fish. Natural variation in cooling and warming includes, beside timing, rate of change on a seasonal scale, and frequency amplitude and rate of change on a short-term, within-season scale. The variation in the latter, i.e. the within-winter stability of temperature, is affected by ice cover. Lake Krankesjön freezes in most winters, but the duration of the ice cover is variable. Other lakes that have more consistent ice cover may show little between-year variation in temperature, but higher variation in duration of low temperatures. However, even in such lakes, fish may experience temperature differences corresponding to the ones explored in our experiment depending on within lake habitat availability. Here, lake morphometry will likely play an important role for the fish's response to low temperature and variable food availability. The thermal habitat of lakes is predicted to change with climate change [59], more so in shallow than in deep lakes [65]. In deep lakes, water temperatures close to the bottom will be close to 4°C, which corresponds to the warmest treatment in our experiment, and prey fish, such as most cyprinids, usually aggregate at greater depths (e.g., [64]). Fish would here be able to migrate to lower temperatures higher in the water column, which would decrease metabolism and, hence, be beneficial in case of food scarcity, but could be connected to a higher predation risk. Corresponding habitat change as a consequence of climate warming has for instance been shown for brown trout in streams [66]. In shallow lakes, such as Lake Krankesjön, the water temperatures can be expected to be rather uniform throughout the lake and under ice-cover close to 0°C. Fish may therefore not be able to select water temperature in order to regulate their metabolism according to the food availability. Instead they may increase their feeding rate, by choosing a habitat with more available food, but often with the trade-off of accepting a higher predation risk [45], [67]. Alternatively, fish may choose to decrease their intake rates during winter and thereby increase their risk of starvation mortality at the benefit of a lower predation risk [68]. In any case, it should be kept in mind that fish may show both individual and species-specific differences in behavioral adaptations for coping with the winter period [45], [69]. Such behavioral differences have potential ecosystem effects through changed feeding rate on lower trophic levels [70] and vulnerability to predation [68].

In conclusion, our results show that the effects of even small temperature differences may be crucial for fish over-winter success and, further, that temperature alone has little power in predicting effects when food abundance is not taken into account. To fully understand the effects of this under field conditions, more work is needed to explore the complex relationship between climate change and zooplankton dynamics, and thus to determine the likelihood and under which conditions that climate change may affect fish populations during winter. We hence suggest that future studies should pay attention to the effects of climate change on over-winter zooplankton biomass and subsequent effects on fish populations.
